# Optimal Method to Stimulate Cytokine Production and Its Use in Immunotoxicity Assessment

**DOI:** 10.3390/ijerph10093834

**Published:** 2013-08-27

**Authors:** Wenchao Ai, Haishan Li, Naining Song, Lei Li, Huiming Chen

**Affiliations:** Chinese Academy of Inspection and Quarantine, Beijing 100123, China; E-Mails: aiwenchao126@163.com (W.A.); lihs@aqsiqch.ac.cn (H.L.); Songnn@aqsiqch.ac.cn (N.S.); lil@aqsiqch.ac.cn (L.L.)

**Keywords:** immunotoxicity assessment, cytokines, whole blood, immunosuppressant

## Abstract

Activation of lymphocytes can effectively produce a large amount of cytokines. The types of cytokines produced may depend on stimulating reagents and treatments. To find an optimal method to stimulate cytokine production and evaluate its effect on immunotoxicity assessments, the authors analyzed production of IL-2, IL-4, IL-6, IL-10, IL-13, IFN-γ, TNF-α, GM-CSF, RANTES and TGF-β in undiluted rat whole blood culture (incubation for 0, 2, 4, 6, 8 or 10 h) with different concentrations of PMA/ionomycin, PHA, Con A, LPS and PWM. We also evaluated the effects of cyclosporin A and azathioprine on cytokine production. The results revealed a rapid increase of IL-2, IFN-γ, TNF-α, RANTES and TGF-β secretion within 6 h after stimulation with 25 ng/mL PMA and 1 μg/mL ionomycin. The inhibition of these cytokine profiles reflected the effects of immunosuppressants on the immune system. Therefore, the results of this is study recommend the detection of cytokine profiles in undiluted whole blood stimulated 6 h with 25 ng/mL PMA and 1 μg/mL ionomycin as a powerful immunotoxicity assessment method.

## 1. Introduction

Cytokines are small molecular weight proteins or peptides secreted by many cell types (particularly immune system cells) that regulate the duration and intensity of the immune response. Cytokines participate in many physiological processes, including the regulation of immune and inflammatory responses. A variety of experiments has shown that either excessive or insufficient production of citokines may contribute significantly to the pathophysiology of a range of diseases. Particularly cytokines released by CD4+ T cells at the onset of an immune response are thought to be decisive for pathological or physiological consequences [1]. Obviously, cytokines play key roles in all immune responses, and offer important new avenues to explore, both in terms of mechanistic understanding of immunotoxicity and of developing new assays to test the immunotoxic potential of the novel compounds [1,2,3,4,5].

Effects on cytokines can be analyzed at the protein level, including direct detection of the relative concentration of various cytokines in the circulation following experimental treatment, and in activated peripheral blood mononuclear cells (PBMCs) or diluted whole blood. Although these assays have (apparently) been used successfully in many instances, there are a number of major problems. First, cytokines generally act primarily at a local level and that they are rapidly cleared from the circulation. Second, cytokines are extremely potent mediators that are active at very low concentrations and expressed only following cellular activation. Finally, for the detection of cytokines in PBMCs or diluted whole blood, the isolation and culture procedures may wash out important immune molecules and xenobiotics, and disrupt molecular networks between immune cells [5,6]. Thus, these methods may be unable to mimic the *in vivo* immunotoxicity environment. On balance, the practical advantages of detection of cytokine levels in stimulated whole blood make it attractive for immunotoxicity screening purposes. Recently, analysis of cytokine production in stimulated whole blood has been used successfully in many instances [7,8,9], since the assay mimics the *in vivo* natural environment. Commonly used stimulating reagents include plant lectins (e.g., PHA, Con A, PWM), LPS, PMA/ionomycin, purified protein derivative of tuberculin (PPD), and anti-CD3 and/or anti-CD28 antibodies. The differences in stimulating reagents and treatments may result in the production of different types of cytokines at different levels. Although the effects of different lymphocyte stimulants on cytokine production had been evaluated in clinical research, there may be differences between species. Unfortunately, there is still no comparative study on the response of cytokine profiles to different stimulants in experimental animals’ whole blood, which makes choosing a suitable stimulation method for nonclinical immunotoxicity evaluation a puzzling task.

Here the authors tested multiple cytokine levels in undiluted rat whole blood after different short-term activations, and evaluated the effects of the immunosuppressants cyclosporine A (CsA) and azathioprine (AZP) on cytokine secretion profiles. Both these drugs are used in patients undergoing organ transplantation and in the treatment of certain autoimmune diseases. CsA has been shown to inhibit mature T lymphocyte activation. It exerts this activity by binding to the cytosolic protein cyclophilin, which is a member of a class of molecules generically known as immunophilins. The cyclosporin-cyclophilin complex targets calcineurin, essentially binding calcineurin to the immunophilin. The calcineurin is then unable to interact with downstream transcription factors (e.g., NF-AT), ultimately preventing the transcription of various cytokines genes [6]. AZP is an analogue of 6-mercaptopurine, which presumably has direct toxicity towards cytokine-producing cells and exerts its pharmacodynamic effects on T cell-mediated immune responses by interference with *de novo* synthesis of purines [10]. The aims of this study were to find a rapid and effective activation method for cytokine production, and provide basic research data and a scientific basis for its application in nonclinical immunotoxicity assessments.

## 2. Materials and Methods

### 2.1. Subjects

Peripheral venous bloods were taken from healthy or immunosuppressant-treated SD rats at 7–8 weeks of age and were collected in vacuum tubes containing dried heparin. Rats were purchased from Beijing Vital River Laboratory Animal Technology Co., Ltd (Beijing, China, licensed by the Beijing Administrative Office of Laboratory Animals).

### 2.2. Stimulating Reagents and Immunosuppressants

PHA, LPS, Con A, PWM, and PMA/ionomycin were all purchased form Sigma, Inc. (St. Louis, MO, USA) and used as stimulating reagents for immune cells. They were diluted with PBS and stored at −4 °C. Rat cytokines multi-plex kits were purchased from Affymetrix, Inc. (Santa Clara, CA, USA).

Two immunosuppressants, CsA and AZP were obtained from Jvshunhong Biological Chemical Co., Ltd. (Wuhan, Hubei, China). Both compounds were dissolved in corn oil as vehicle.

### 2.3. Whole Blood Stimulation

Heparinized whole blood was taken from healthy rats and added in 96-well plates (200 μL/well), supplemented with different concentrations of each stimulating reagent (PMA: 1, 5, and 25 ng/mL; Ionomycin: 1 μg/mL; PHA, LPS, Con A, PWM: 1, 5, and 10 μg/mL), and incubated for 0, 2, 4, 6, 8 or 10 h at 37 °C with 5% CO_2_. After incubation, the bloods were centrifuged and supernatants were collected. The supernatants were stored at −80 °C prior to the detection of cytokines.

### 2.4. Cell Viability Test

A total of 1.0 × 10^6^ PBMCs from indicated treatment blood was collected after Ficoll-Hypaque density gradient centrifugation, and washed twice with ice-cold phosphate-buffered saline (PBS). Cells were then stained with fluorescein isothiocyanate (FITC)-labeled annexin V and propidium iodide (PI) according to manufacturer instructions (Invitrogen, Carlsbad, CA, USA) and then subjected to flow cytometric analysis (Beckman Coulter, Fullerton, CA, USA).

### 2.5. Effects of Immunosuppressants on Cytokines Profiles

Rats were treated with CsA or AZP by daily gastric intubation for seven consecutive days. The dosing volume was 1 mL/kg body weight. Seven groups of three rats each were exposed to 1.25, 5, and 20 mg CsA/kg body wt/day, or 1, 5, 25 mg AZP/kg body wt/day, or vehicle (olive oil), respectively. The high dose of each immunosuppressant was maximum tolerated dose (MTD) [10,11]. Peripheral venous bloods were taken from all rats on Day 7 and were activated using selected method. The supernatants of blood cultures were collected and were stored at −80 °C prior to the detection of cytokines.

### 2.6. Cytokines Determination by the Multi-Plex Technology

The supernatants were analyzed simultaneously for 10 cytokines, including IL-2, IL-4, IL-6, IL-10, IL-13, IFN-γ, TNF-α, GM-CSF, RANTES and TGF-β with a Bio-Plex machine, which employs a bead-based sandwich immunoassay. For the detection of multiple cytokines, a monoclonal antibody specific for each cytokine was coupled to a particular set of beads with known internal fluorescence. Multiple cytokines antibody-coated beads were pooled together to allow the cytokines to be measured simultaneously. The assay was performed according to the manufacturer’s instructions (Bio-Rad Laboratories, Richmond, CA, USA) and analyzed with the Bio-Plex Manager software (version 4.0). The sensitivity of this method was lesser than 10 pg/mL and the assay could accurately detect cytokines in the range of 1–32,000 pg/mL.

### 2.7. Statistical Analyzes

All data were presented as mean ± sd (*x* ± *s*). Analysis of variance (3-way or one-way ANOVA) was used to compare values among all groups. Statistical analysis was undertaken with SPSS 10.0 (SPSS, Inc., Chicago, IL, USA) and *p* value of <0.05 was considered as statistically significant.

## 3. Results

### 3.1. *In Vitro* Detection of Cytokines

**Figure 1:**
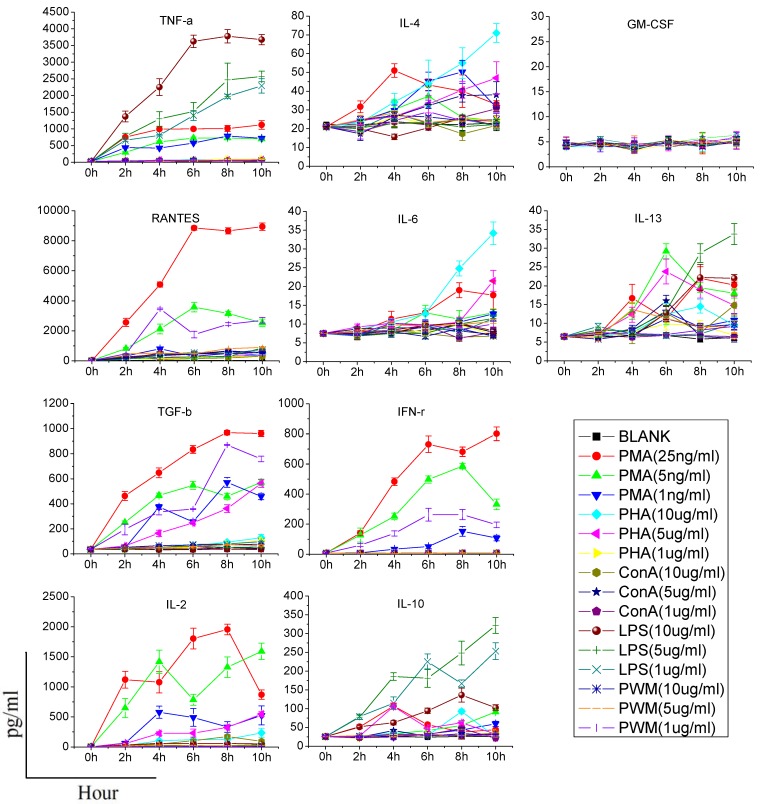
Rat blood immune cells show altered cytokine production in response to different stimulants.

The results showed that each stimulant induced production of a unique cytokine profile (Figure 1). LPS significantly (*p* < 0.01 or *p* < 0.05) increased the productions of TNF-α, RANTES, and IL-10 within 2 to 6 h and weakly promoted production of IL-2 in 25 μg/mL group. PHA increased (*p* < 0.05) the productions of IL-2, RANTES and TGF-β after 8 h of stimulation. PHA also weekly increased the levels of IL-4 and IL-6. PWM (1 μg/mL) significantly (*p* < 0.05) increased the productions of IFN-γ, RANTES and TGF-β after 6 h. The PMA/ionomycin combination significantly increased (*p* < 0.01 or *p* < 0.05) the production of IL-2, IFN-γ, TNF-α, TGF-β and RANTES within 4 to 6 h. In contrast, Con A had lesser effects on cytokine production as it only increased significantly (*p* < 0.05) the level of RANTES. Taken together, stimulation with 25 ng/mL PMA and 1 μg/mL ionomycin for 6 h was chosen as the method for the remaining experiments, in light of their ability to produce maximum amounts of cytokines in a short time.

### 3.2. Cell Viability Test

Immune cell viability was measured by flow cytometry after 6 h treatment by PMA/ionomycin. The results showed that the percentages of double negative cells, with or without stimulation, were 84.68% and 86.17%, respectively, indicating that immune cells cultured in the whole blood were alive for more than 6 h (data not shown).

Values are presented as M ± SD (n = 3).

### 3.3. Immunosuppressants Dose-Dependently Inhibited the Secretion of Cytokines

**Figure 2:**
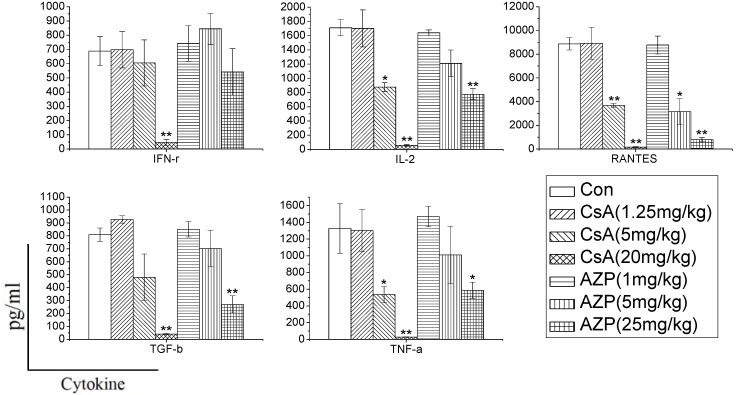
Cytokine secretions were inhibited by different immunosuppressants.

After seven days of oral administration with CsA, AZP or vehicle, peripheral venous blood was taken from each rat. Cytokine profiles in whole blood were measured after stimulation *in vitro* with 25 ng/mL PMA and 1 μg/mL ionomycin for 6 h. As shown in Figure 2, the levels of IFN-γ were lower (*p* < 0.01) in 20 mg CsA/kg group than the other groups. The levels of IL-2, RANTES, TGF-β and TNF-α were significantly (*p* < 0.05 or *p* < 0.01) decreased in the 5, 20 mg CsA/kg and 5, 25 mg AZP/kg groups in comparison with those of control groups. The results stated above suggested that the influence of an immunosuppressant on the amount of cytokine secretion is proportional to the administered dose of that drug. The changes in cytokine secretion profiles might thus be able to reflect the effects of immunosuppressants on the function of immune cells.

Values are presented as M ± SD (n = 3) (* *p* < 0.05, ** *p* < 0.01, as compared with control).

## 4. Discussion

Cytokine production is one of the first steps of the immune response and can provide important information regarding the nature of any immunotoxic responses. As resting immune cells only produce a minimal amount of cytokines to meet their basic cellular requirements, cytokine profiles from inactivated blood might not accurately reflect the immune function status. Stimulation is necessary for the measurement of cytokine production. However, it is difficult to select an appropriate stimulation method, so we measured the production of multiple cytokines by *in vitro*-stimulated immune cells in undiluted rat blood and evaluated the effect of immunosuppressants on cytokine profiles.

Due to the highly pleiotropic and redundant nature of cytokines, a single biological event can be affected by the complex interplay between several cytokines, so the most important thing is to find which cytokine is “appropriate” for immunotoxicology assessment. From previous studies, the most useful cytokines can be divided into the following groups [6]: (1) pro-inflammatory cytokines (e.g., IL-1, IL-6, TNF); (2) TH1 and TH2-type cytokines, which include IL-2, IL-4, IL-10, and IFN-γ, TGF-β; (3) IL-1 receptor antagonist (IL-1RA); (4) chemokines (e.g., IL-8, MIP, RANTES); (5) hematopoietins (e.g., GM-CSF, IL-3); (6) type I interferons (IFN-α, IFN-β), type II interferons (IFN-γ); (7) others (e.g., IL-9, IL-13, IL-14, oncostatin M, leukemia inhibitory factor, and leukoregulin). Since the number of items that can be tested using rat cytokine multiplex kits is limited, we tested IL-2, IL-4, IL-6, IL-10, IL-13, IFN-γ, TNF-α, GM-CSF, RANTES, and TGF-β from among these useful cytokines.

When assessing cytokine production by stimulants, two points should be made. First, the stimulant used should be titered to the lowest concentration that results in cellular activation; otherwise, it is possible that modest alterations in cytokine production may be masked. Second, the time of stimulation should as short as possible, since the function of immune cells may potentially be affected once they are removed from the animal [6]. Consequently, we need to find an effective stimulant which can promote a maximum amount of cytokine production at a lower dose and in a shorter time. In our study, although PHA, LPS, Con A, and PWM increased the production of some cytokines in a dose or time dependent manner, their effects were limited or time-consuming. PMA/ionomycin was the optimal stimulant in our study, and could significantly increase production of five cytokines in 6 h without any significant damage to immune cells. PMA activates protein kinase C, while ionomycin is a calcium ionophore, and stimulation with these compounds bypasses the T cell membrane receptor complex and will lead to activation of several intracellular signaling pathways, resulting in T cell activation and production of a variety of cytokines. Many clinical studies have also confirmed that PMA could specifically increase the production of certain cytokines (especially TH1-type cytokines). Barten *et al*. have reported on an increased IFN-γ, TNF-α, IL-2, IL-6 production in whole blood from healthy people stimulated 5 h by 25 ng/mL PMA and 750 ng/mL ionomycin, while the IL-4 and IL-10 levels were not affected [12]. Keski-Nisula *et al*. found that after 24 h culture, PMA/ionomycin increased the secretion of IFN-γ, IL-6 and TNF-α in diluted whole blood from healthy people, but not IL-4 [13]. In another study, PMA was used to stimulate human CD4+ T cells and production of IL-4, IFN-γ, IL-17 and IL-10 was analyzed. They also found that there was no IL-10 production [14]. In addition, contrary to the result of previous research [6], our study did not find any significant effect of PHA on promotion of the production of IFN-γ, IL-10, IL-4, IL-6 and TNF-α, and only increases of IL-2, IL-13 and RANTES were observed. Differences in species and experimental conditions may explain this discrepancy.

In order to characterize whether different immunosuppressants have distinct effects on cytokine production, and determine whether the effects are dependent on the dose, we used CsA and AZP as model compounds with known immunotoxic activity. Rats were administrated the two drugs to mimic immune system damages in the whole body. In the study, we found that the two immunosuppressants dramatically inhibited the cytokine production ability dose dependently. CsA significantly inhibited the production of most cytokines (IFN-γ, IL-2, RANTES, TGF-β, TNF-α), while AZP had a weak influence on the production of IFN-γ. Previous research has also found that dexamethasone (DEX), tacrolimus (FK506) and mycophenolic acid (MPA) have distinct effects on the release of cytokines. DEX nonspecifically and dose-dependently inhibited the production of 12 cytokines [7]. In contrast, FK506 and MPA selectively inhibited the secretion of IL-2 and IL-13. Therefore, changes in cytokine production in whole blood culture may reflect the unique signature of each xenobiotics, which are conducive to identify their toxicity on the immune system.

## 5. Conclusions

According to the results of this study and the aforementioned discussion, it is concluded that stimulation of undiluted whole blood with 25 ng/mL PMA and 1 μg/mL ionomycin for 6 h in is an effective method to produce a variety of cytokines. The inhibition of these cytokine profiles reflected the effects of xenobiotics on the immune system. Therefore, simultaneous detection of cytokine profiles in undiluted whole blood stimulated with PMA/Ionomycin using a multi-plex technique is a simple, rapid and sensitive tool for immunotoxicity assessment.
